# The determinants of common bean variety selection and diversification in Colombia

**DOI:** 10.1016/j.ecolecon.2021.107181

**Published:** 2021-12

**Authors:** Hernan Botero, Andrew P. Barnes, Lisset Perez, David Rios, Julian Ramirez-Villegas

**Affiliations:** a Postdoctoral Researcher in the Rural Economy (REES), Environment and Society Research Group, Kings Buildings Campus, West Mains Road, Edinburgh EH9 3JG, UK; b Head of Department of Rural Economy, Environment and Society (REES), SRUC, Kings Buildings Campus, West Mains Road, Edinburgh EH9 3JG, UK; c Researcher in International Center for Tropical Agriculture (CIAT), Km 17 Recta Cali-Palmira, 763537 Cali, Colombia; d CGIAR Research Program on Climate Change, Agriculture and Food Security (CCAFS), c/o CIAT, Cali, Colombia; e Bioversity International, Rome, Italy; f Plant Production Systems Group, Wageningen University, Wageningen, The Netherlands; g University of Copenhagen, Copenhagen, Denmark

**Keywords:** Farm input markets, Land use, Global warming, Choice models, Classification methods

## Abstract

Variety selection and diversification are climate change adaptation practices pursued by Colombian common bean producers. We investigate the drivers behind common bean variety selection and diversification in one of the most important common bean production regions in Colombia —Santander. The effects of climate change on this region are expected to be elevation driven. Exploiting the relationship between elevation-driven weather variations and climate change perception in Santander, we estimate an alternative-specific conditional logistic regression model to identify the determinants of common bean variety selection from a survey of producers. Using an ordered-logistic regression model, we also investigate the drivers behind common bean variety diversification within this farming community. We find that farms' elevation, household composition, and seed certification are some of the most important drivers behind farmers' common bean variety selection in Santander. We also find that varieties that sell at higher prices and have shorter vegetative cycles tend to be more preferred by farmers. Finally, farmers who receive more help from family members and own a tractor tend to grow more than one variety in the same production cycle. Common bean breeding programmes can exploit these drivers to design communication strategies to maximize uptake of newly developed common bean phenotypes.

## Highlights

• Variety- and farmer-specific determinants of common bean variety selection and diversification in Colombia are identified.
• An alternative-specific conditional logistic regression model is used to identify the drivers behind variety selection.
• An ordered logistic regression model is used to identify the drivers behind common bean variety diversification.
• Variety selection depends on farms' elevation and household composition, plants’ life cycle length, and market price.
• Diversification depends on household composition, farms' geographical location, and farmers' ownership of capital goods.

## Graphical abstract

**Figure:**
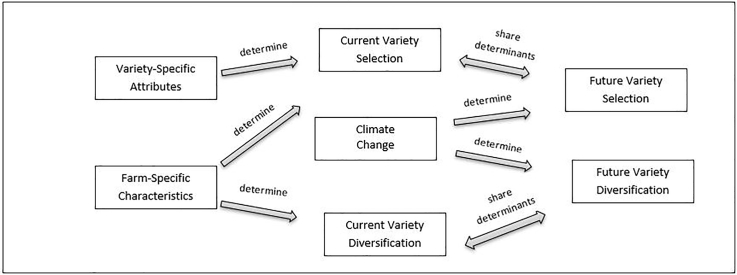

## 1 Introduction

Colombian weather patterns are elevation-driven and strongly influenced by El Nino Oscillation (ENSO) (Cepal, 2012; IPCC, 2014; Buhr et al., 2018). El Niño phase tends to generate higher daily temperatures, less daily precipitation, and more recurrence of droughts, especially in low-elevation geographical areas. In contrast, la Niña phase tends to generate lower daily temperatures, more daily precipitation, and more instances of flood and extreme temperature variations, especially in high-altitude geographical areas (Poveda and Mesa, 1996; Poveda et al., 2011; Henao et al., 2020). This weather instability generates an asymmetric impact on agricultural productivity. While el Niño phase increases the prevalence of abiotic stresses in crops, la Niña phase increases the prevalence of biotic stresses and the risk of crop destruction due to floods and landslides (Santos, 2006; Duque et al., 2013; IDEAM, 2013). These patterns are expected to get worsened by climate change, negatively impacting 60% of the current Colombian agricultural production areas and 80% of the crops that Colombian farmers currently cultivate (Feola and Binder, 2010; Ramirez-Villegas et al., 2012; Eitzinger et al., 2014; IPCC, 2014).

Climate change is expected to particularly affect the production of common beans (*Phaseolus vulgaris*) in Colombia. Some of the traditional common bean varieties cultivated by Colombian farmers, such as Calima, are highly vulnerable to extreme temperatures and reduced levels of rainfall (Schoonhoven and Voysest, 1991; CIAT, 2008; Troyo-Diéguez et al., 2010). Consequently, a worsened ENSO is expected to reduce the profitability of the cultivation of traditional common bean varieties (CIAT, 2008). In addition, low-elevation geographical areas are expected to become less suitable for the cultivation of traditional varieties of common beans because they will have an increased prevalence of biotic and abiotic stresses with a worsened ENSO (Ramirez-Villegas et al., 2012; Eitzinger et al., 2014; Güiza-Villa et al., 2020). Finally, producers located at higher elevations have a limited capacity to adapt to climate change since a hilly topography limits the use of heavy machinery or bulky technology to cultivate common beans (Ramirez-Villegas et al., 2012; Feola et al., 2015; Acevedo and Martinez, 2016).

There are several adaptation strategies that Colombian common bean growers can pursue at farm-level (Smit and Skinner, 2002; Clements et al., 2011; Asfaw et al., 2013a, Asfaw et al., 2013b; Niles et al., 2015; Islam et al., 2020). One of the most common adaptation strategies proposed for this farming community is the use of improved seeds (Hailu et al., 2015). There are several companies supporting common bean breeding programs in Colombia (Schoonhoven and Voysest, 1991; Blair, 2003; Muñoz et al., 2008; CIAT, 2008; Hershey and Neate, 2013). This work has mainly focused on making common beans more resistant to the most acute and prevalent biotic stresses present in Colombia (Leon and Jimenez, 1997; Leon and Jimenez, 2002; FENALCE, 2011; Beebe et al., 2011). However, the uptake of these new varieties has been low and Colombian farmers continue to grow traditional varieties, which are mostly exchanged in informal or non-market settings (Sperling and McGuire, 2010; FENALCE, 2020).

The development of common bean varieties in Colombia has been mainly based on expert opinion about the needs of farmers, partially disregarding the determinants of farmers' demand for particular common bean attributes (Chauhan et al., 2020; Jochua et al., 2020; Ribeiro et al., 2020). Some studies in Africa have shown that demand depends on agronomic and economic attributes and farmers' socioeconomic characteristics (Katungi et al., 2011; Katungi et al., 2015; Sichilima et al., 2016). To our knowledge, no research has been performed on identifying the drivers behind farmers' demand for the attributes of common beans in a Latin American context. According to the international evidence, farmers' demand for phenotypes under development by plant breeding institutions depends on factors that also help determine the demand for current commercial phenotypes (Sichilima et al., 2016; Shikuku et al., 2017; Vanegas, 2017; Onzima et al., 2019). These factors are also expected to influence farmers' response to extension and commercial programs that promote the voluntary uptake of the new varieties. Consequently, identifying the factors that determine farmers' demand for common bean varieties allows agricultural companies, extension service providers, and seed suppliers to create commercial and communication strategies aimed at maximizing the uptake of the new varieties under development (CIAT, 2008; Sichilima et al., 2016; Eitzinger et al., 2018). This is particularly relevant to Colombia where farmers' demand for common beans is expected to be influenced by the elevation in which farms are located, which implies that seed suppliers and companies in charge of common bean breeding programs should consider elevation as an important factor to develop new varieties and design commercial and engagement strategies.

Consequently, the aim of this paper is to provide the first identification of the factors that determine Colombian farmers' demand for common bean varieties. By analysing the responses to a revealed-preference survey of 566 common bean producers in the department of Santander, this paper performs the first econometric estimation of the determinants of the demand for common bean varieties in Colombia. These determinants are identified employing an alternative-specific conditional logistic regression model. In addition, this paper also performs the first identification of the factors that determine variety diversification in Colombia using an ordered logistic regression model.

The department of Santander has been selected for this study because it is the fifth most important common bean producing region in Colombia (DANE, 2014) and it is expected to be the worst affected by climate change among the most important common bean production regions in Colombia (Ramirez-Villegas et al., 2012; Eitzinger et al., 2014; Eitzinger et al., 2018). Moreover, Santander's municipalities are mainly located on the Andean mountains, which implies that Santander has the archetypal mountainous landscape of the Colombian Andes and any inference based on this region is easily extendable to other Colombian regions with similar agroecological environments and elevation-driven weather variations (Perez et al., 2019; Botero et al., 2020).

## 2 Conceptual framework

Valuation methods applied to the stated-preference exercises estimate farmers' willingness to pay for common beans' attributes and the magnitudes of the trade-offs that farmers are willing to accept to exchange one attribute for another (Katungi et al., 2011a; Katungi et al., 2015). Stated-preference experiments have been employed to measure farmers' preferences for agricultural products' and seeds' attributes in different parts of the world (Drucker and Anderson, 2004; Asrat et al., 2010; Mahadevan and Asafu-Adjaye, 2015; Sánchez-Toledano et al., 2017; Acheampong et al., 2018; Jin et al., 2020). This methodology has been used extensively in Africa to determine consumers' and farmers' demand for the attributes of common beans (Lambrecht et al., 2013a, Lambrecht et al., 2013b; Lambrecht et al., 2015; Assete et al., 2018). One important advantage of stated-preference experiments is that the experimenter may manipulate the attributes offered to consumers and farmers to study their willingness to pay for each attribute based on their variety selection. Its most important disadvantage in agricultural settings is that experiments are usually applied to varieties that are not in the market yet, which impedes the experimenter to utilise the market value of the options offered. Consequently, these studies usually rely on hypothetical economic values and rewards to elicit behaviour, which may have important consequences on the consistency of the answers (Kuhberger et al., 2002; Locey et al., 2011; Luchini and Watson, 2014).

Revealed-preference or market methods are used as an alternative approach to stated-preference experiments (Louviere et al., 2000). In contrast to stated-preference experiments, revealed-preference methods do not rely on controlled experiments to obtain information on variety selection but on actual input choices. This methodology can be used to estimate the determinants of variety selection based on the actual seed choices made by commercial farmers. It utilises market information on variety choices to draw conclusions on the factors that determine the common bean variety selection observed in the market. Apart from differing in the source of information employed, revealed- and stated-preference methodologies utilise identical estimation methods and their estimated parameters have similar interpretations. This implies that both methodologies are able to determine a ranking of preference for common bean varieties, with the only difference that one relies on hypothetical selections and the other on market ones.

The main drawback of the revealed-preference method is that market choices do not include the whole universe of potential choices available to farmers, whereas in stated-preference experiments all existing varieties may potentially be included. Consequently, conclusions resulting from a revealed-preference estimation only apply to the varieties actually selected in the market, whereas the conclusions drawn from stated-preference experiments apply to the whole sample of varieties presented to farmers during the experiment. The main advantage of the revealed-preference method is that information on farmers' socioeconomic characteristics is more reliable since this information is collected through face-to-face interviews with commercial farmers, which usually takes place in the farms where common beans are grown. In contrast, farmers' socioeconomic information collected through stated-preference experiments depends on farmers' willingness to participate in the experiments, which in turn depends on transportation costs and farms' distance to the study site where experiments usually take place, generating a sample selection bias that may potentially affect the generalization of the results drawn from these experiments. As a result, the revealed-preference methodology tends to do a better job in identifying farmer-specific determinants of variety selection than the stated-preference one, but a poorer job in identifying variety-specific determinants (Louviere et al., 2000; Katungi et al., 2011a; Katungi et al., 2015).

In this study, we employ a revealed-preference approach to identify the factors that determine variety selection of common beans in Colombia. We further employ an ordered logistic regression model to investigate the drivers behind seed diversification. Following to Katungi et al. (2015), we use a combination of variety-specific and farmer-specific characteristics as the determinants of variety selection. Following to Onzima et al. (2019), we use a set of farm-specific factors as the explanatory variables of seed diversification. Two important determinants of variety selection introduced in this study are farms' elevation and distance to the nearest urban centre. According to Feola et al. (2015), future weather variations in Colombia are expected to be elevation-driven, which will have a differentiated effect on common bean production regions. Ramirez-Villegas et al. (2012) estimate that high-elevation farms will experience more extreme temperature variations and unpredictable seasons and low-elevation farms will experience more droughts and lower rainfall levels. Botero et al. (2020) find that elevation is an important driver of climate change perception in this region of Colombia. Farmers located at low elevations tend to perceive more droughts and water deficits, whereas farmers located at high elevations tend to perceive extreme temperature variations, even though they consider that they have enough water for their bean production. As a result, farms' elevation is expected to be an important driver behind farmers' variety selection. In turn, farms' distance to the nearest urban centre is expected to be an important driver behind variety selection because distance determines farms' accessibility in the Andean mountains (Feola et al., 2015; Botero et al., 2020), affecting farmers' transportation costs. In turn, these two variables are also expected to be important determinants of seed diversification. Elevation is expected to reduce seed diversification because it is more complicated to cultivate several crops in a steep land field. Distance is also expected to reduce seed diversification because more varieties grown imply a more spatially scattered demand, increasing transportation costs (Feola et al., 2015; Eitzinger et al., 2018). The whole set of regressors utilised in this study is introduced and explained in detail in the next section.

## 3 Data and descriptive analysis

### 3.1 The study site

According to IPCC (2014), the northeast of Colombia will be the most affected region with climate change. Santander is selected for this study because this region is expected to be the most affected common bean production area in Colombia and it is one of the most affected areas by ENSO (Ramirez-Villegas et al., 2012; Eitzinger et al., 2014; IPCC, 2014). Most municipalities in Santander have a hilly topography because they are located along the eastern side of the Colombian Andes. In Colombia, temperature and rainfall variations are elevation-driven. Low-elevation farms tend to have higher temperatures and lower rainfall levels and high-elevation farms tend to have lower temperatures and higher rainfall levels. This elevation-driven weather variation also affects the types of products grown in each thermal floor^1^ and the varieties grown of the same crop (IDEAM, 2013; Eitzinger et al., 2018; FENALCE, 2020).

**Fig. 1:**
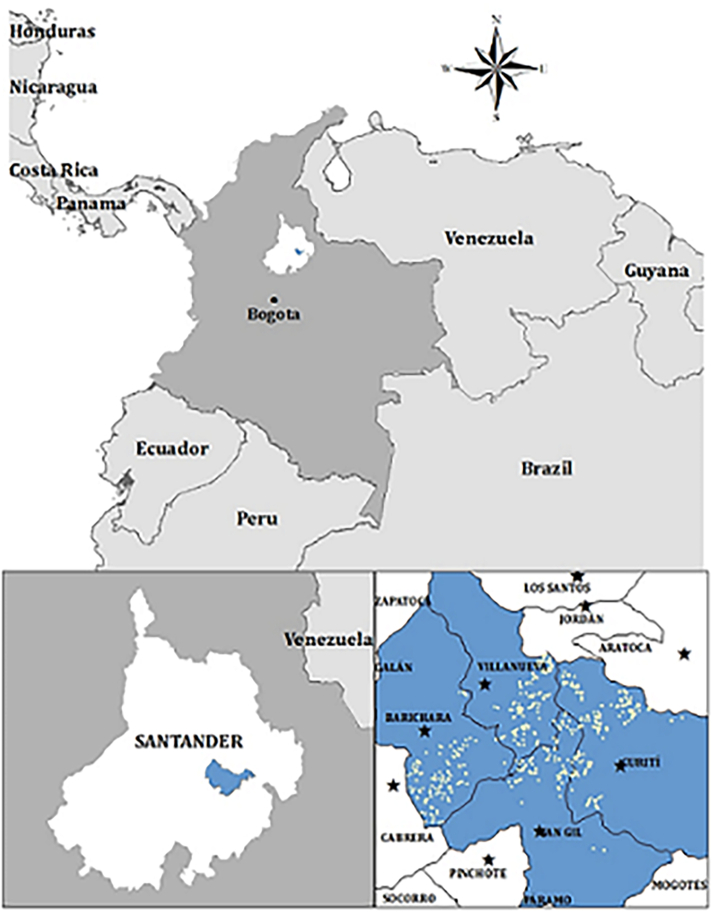
Geographical distribution of flames.

Four municipalities are selected for this study: Barichara (6.6358° N, 73.2234° W), Curití (6.6063° N, 73.0687° W), San Gil (6.5548° N, 73.1341° W), and Villanueva (6.6709° N, 73.1748° W). Two criteria were used to select the study area. First, these municipalities are among the most important common bean production areas in Santander according to the 2014 Colombian Agricultural National Census. This allows having in the sample farmers with extensive knowledge on common bean production and on adaptation strategies to tackle the negative effects of ENSO on common bean production. Second, these municipalities have different elevations, which results in a different temperature and rainfall level depending on the elevation in which farms are located. Barichara (with an average elevation of 1266 masl) and Villanueva (1288 masl) tend to have higher temperatures and less rainfall than Curití (1568 masl) and San Gil (1452 masl). Consequently, common bean variety selection is expected to depend on each municipality's elevation. Fig. 1 in appendix shows a map of the study site.

Perez et al. (2019) explains in detail the questionnaire constructed for this study. The information was collected by CIAT and CGIAR as part of “AgroClimas” project, which is aimed to understand common bean growers' decision-making at farm level in areas highly affected by ENSO (Rios et al., 2017). This paper focusses on the section related to common bean variety selection. The survey was run during August/September in 2017 and the sample includes 572 common bean growers who operate in the region. To maximize the response rate, interviewers visited all farms known to grow common beans in these four municipalities to carry out face-to-face interviews. Out of the 572 interviews performed, only 566 farmers provided useable information for this study. The survey adopts a livelihoods approach (Scoones, 1998) and collects information on farmers' human, economic, financial, and physical capitals, and on household composition. It also collects information on input and land use, focusing particularly on common bean variety selection and land use under beans.

### 3.2 The Survey: some descriptive statistics of the study site

Table 1 in the appendix presents information on the varieties grown in the study site and the number of farmers who grow each these varieties. This table also shows the distribution of farmers per the number of common bean varieties grown in the same production cycle. Table 2 in the appendix presents the attributes of each common bean variety grown. The information contained in Table 1, Table 2 indicate that Santander farmers grow only eight varieties of common beans: Calima, Froilan, Corpoica JiJi, Zaragoza, Carbabello, Radical, Bola Roja, and Cargamanto Rojo. The most popular common bean variety among Santander farmers is Radical followed by Cargabello and Zaragoza, and the least popular varieties are Corpoica Jiji and Cargamanto Rojo. This low variability for some of varieties surveyed may affect the efficiency with which parameters are estimated, but it does not affect its unbiasedness (Louviere et al., 2000; page 263). We estimate the econometric model proposed below using a white variance-covariance matrix in order to reduce the heteroscedasticity that this low variation in responses for some of the varieties causes. In addition, 94.2% of the sample grows only one variety of common beans, 5.13% of the sample grows two varieties, and 0.71% of the sample grows three varieties in the same production cycle. There is no farmer in this region that grow four or more varieties simultaneously. In turn, 7 out of 8 varieties grown are bush varieties with a determinate growing habit^2^ and only one variety —Cargamanto Rojo— is a climbing plant with an indeterminate growing habit. Moreover, there are four varieties that have a solid red colour (Froilan, Corpoica Jiji, Radical and Bola Roja), three have a mottled red colour (Calima, Cargabello, and Cargamanto Rojo), and only one variety has solid light pink colour (Zaragoza). Another attribute considered is the potential yield per hectare. This information was taken from Federación Nacional de Cultivadores de Cereales, Leguminosas y Soya (FENALCE) and Corporación Colombiana de investigación Agropecuaria (AGROSAVIA)’s websites for the varieties they offer, which is complemented with the information provided by Schoonhoven and Voysest (1991) for this attribute. In Table 2, vegetative refers to the number of days required by the plants to reach the flowering phase starting from seeds, size refers to the average seed weight in gr, and shape refers to the categorization utilised by CIAT to define seeds' physical appearance.^3^ According to Katungi et al. (2015), these attributes are among the most important determinants of farmers' variety selection in Africa.

**Table 1:** Distribution of farmers per common bean variety grown and per the number of varieties simultaneously grown in the same season (*N* = 566).

Varieties Grown	Number of Farmers	Number of Varieties Grown	Number of Farmers
Calima	31	1	533
Froilan	35	2	29
Corpoica Jiji	2	3	4
Zaragoza	58		
Cargabello	81		
Radical	391		
Bola Roja	3		
Cargamanto	2

**Table 2:** Attributes of the Common Bean Varieties Grown in Santander, Colombia

ID	Name	Size	Shape	Colour	Habit	Potential yield/ha	Vegetative	Average yield/ha	Average price/kg
1	Calima	0.48	long oblong	mottled red	I	1800	100	1145	3108
2	Froilan	0.56	short oblong	solid red	I	1693	95	1215	3064
3	Corpoica Jiji	0.53	rounded	solid red	I	1657	95	813	3080
4	Zaragoza	0.5	ovate and kidney-shaped	solid light pink	I	2100	90	1017	3343
5	Cargabello	0.42	short oblong	mottled red	I	1200	94	1075	3111
6	Radical	0.5	short oblong	solid red	I	1884	98	1108	3082
7	Bola Roja	0.65	rounded	solid red	I	2600	200	1250	2892
8	Cargamanto	0.72	long oblong and kidney-shaped	mottled red	III	2600	165	1072	3118

Table 3 in the appendix presents the descriptive statistics of the explanatory variables of common bean variety selection and diversification chosen for this study. Farms' elevation is an important variable collected. In our sample, the median elevation value is 1573 masl. The distribution of elevation is symmetric around its median value, with a minimum value of 1264 masl and maximum value of 2014 masl. Another important variable collected is farms' distance to the urban centre of the municipality in which they are located/registered. Almost all common bean producers surveyed are located near to these urban centres. The distribution of distance is also symmetric around its median value of 5.14 km, with the nearest farm located at 2.03 km from its municipality's urban centre and the most distant one is located at 8.16 km.

**Table 3:** Descriptive Statistics of the Explanatory Variables (N = 566).

Variables	Statistical Summary						
	Avg.	Std. Dev	Min.	1st Percentile	Median	3rd Percentile	Max.
yield/ha (kg)	1096.40	382.81	125	833	1000	1406	2000
price/kg (CO$)	3101.38	378.46	1680	2956	3042	3253	4000
household head's age (years)	49.053	13.53	17	38	49	59	82
household head's years of education	4.52	3.23	0	2	4	5	18
farm area (ha)	2.02	1.91	0.15	1.00	1.50	2.50	21.00
elevation (masl)	1581	119	1264	1508	1573	1658	2014
female importance (females - males)	−0.11	1.32	−4	−1	0	1	5
distance (km)	5.13	1.25	2.03	4.27	5.14	5.91	8.16


Variables	Number of Farmers, Inhabitants, and Varieties							
	1	2	3	4	5	6	7	8
number of inhabitants in the farm (#)	21	119	125	133	108	38	16	6
number of crops cultivated (#)	349	159	38	14	3	2	1	
number of farmers living in the farm (#)	303	177	53	25	5	3		


Variables	Possible Answers	
	0	1
gender (1 = male, 0 = female)	107	459
certified seeds (1 = certified, 0 = otherwise)	556	10
access to less rainfall (1 = yes, 0 = no)	146	420
own a tractor (1 = yes, 0 = no)	558	8

Another expected driver of variety selection and diversification is farm size. Almost 70% of the farms in our sample are smaller or equal to 2 ha, which is the average farm size in the Colombian Andean mountains (Perfetti et al., 2013), and only 2% of the farms surveyed are larger than 10 ha. As a result, common bean growers in this Colombian region are mainly small scale, low-income farmers with small land areas to cultivate crops. Another potential driver of common bean selection and diversification considered is the number of years of formal education of the household head. In our sample, household heads have an average of only 4.5 years of formal education, 75% of the sample ended primary school (5 years of formal education), though 2% have an undergraduate degree (16 years of formal education).

Household head's age is a proxy for farmers' experience with common bean production (Hassan and Nhemachena, 2008; Ainembabazi and Mugisha, 2014; Dhananjaya and Thayaparan, 2016). In our sample, the age distribution is symmetric around its median value of 49 years old. The youngest household head surveyed was 17 years old and the oldest 82. This age distribution is representative of the national age distribution, which is also symmetric around its mean value of 58 years old and has a minimum value of 13 and maximum of 99 (DANE, 2014). In turn, we use two variables to investigate the effect of females on common bean production decisions. On the one hand, we estimate the importance of females in the household composition. This variable is computed as the number of females living in the farm minus the number of males living in the farm. A value of 0 for this variable implies that there are as many women in household as men. A positive value implies that there are more women than men. In our sample, the median value for the importance of females in the household is zero, which implies that in 50% of the households there are more women than men and in the other 50% there are more men than women. On the other hand, we also consider the number of household heads that are men. This variable is constructed as 1 if the household head is male and 0 otherwise. In our sample, a male is the household head in 81% of the households surveyed. We use two gender variables because one of estimation methodologies used in this study performs poorly with dichotomous regressors, which is corrected with the variable of female importance defined here.

Another important limiting factor that affects the decision of farmers to grow common beans in tropical zones is whether they have access to a stable water source (Beaver et al., 2010; Castro-Guerrero et al., 2016; Le-Roux et al., 2018). Common bean plants require a stable water source during its lifecycle, especially in the initial vegetative phase and during flowering, when the beans are produced (Castro-Guerrero et al., 2016). As a result, variety selection and diversification are expected to be affected by farmers' access to a stable water source. We consider water supplied from the aqueduct as a stable water source since this service is provided by the government, which may use larger resources to keep its provision stable. In our sample, 74.2% of the farms have access to the aqueduct. In addition, not all farms in our sample have a water stream nearby to use as a stable water source. In fact, only 15% of the sample indicates to have a water stream nearby, but none of these farmers use that source for common bean production. Consequently, the aqueduct becomes the most stable water source in the region and the main source apart from precipitation.

An important limiting factor for agricultural production in Colombia is that input markets are geographically segmented (Mendola, 2005). Farmers usually have a limited supply of workers, forcing them to do most of the farm work themselves or rely on family help. This implies that the separability condition between consumption and production decisions does not apply (Singh et al., 1986; Mendola, 2005), which implies that Colombian farmers' production decisions are dependent on household composition. We use the number of farmers living in the farm and the total number of inhabitants of the farm to control for household composition. In our sample, the most common household composition is four inhabitants living in the farm, followed by three and two inhabitants living in the farm, respectively. In turn, the most common household composition regarding the number of farmers living in the farm is one farmer, followed by two and three farmers, respectively. In our sample, there is no household that has more than eight inhabitants or more than six farmers living in the farm.

Common beans are usually intercropped with maize, tobacco, or coffee in Colombia (Santalla et al., 2001; Iannetta et al., 2013; Kumar-Singh et al., 2013; Harelimana et al., 2018). Intercropping can be practiced as a way of diversifying the number of crops grown in the farm to generate more income sources. Intercropping is better performed with certain varieties of common beans, such as Calima or Radical (Iannetta et al., 2013). As a result, the number of crops grown are also expected to affect variety selection. We use the number of crops grown to control for intercropping practices. In our sample, 61.66% of the sample only grows common beans, 28.1% grows beans and another crop, and nearly 10% of the sample grows two or more crops apart from common beans.

Seed certification is expected to affect variety selection. Utilising certified seeds is not a common practice among Santander farmers. Only 10 farmers in our sample used certified seeds for their common bean production cycle of August/September of 2017: Radical (8), Cargamanto Rojo (1), and Calima (1).^4^ The rest of the sample relied on seeds saved from previous production cycles, given by a friend or relative, or bought in an informal transaction from a neighbour. Another important restriction confronted by this common bean producers is whether they own a tractor for land preparation. We collected information on the number of farmers who own a tractor, finding that 1.41% of the sample owned one. This seems characteristic of common bean producers on the Andean mountains because tractors are hard to utilise on a very steep landscape, and farmers prefer to rely on manual or animal-pulled devices to prepare the land for cultivation.

Two final variables that are expected to be important drivers behind variety selection are yield per hectare and price per kg. Even though these values are not known before common bean varieties are selected, farmers with experience on common bean production are expected to (imperfectly) forecast both the yield per hectare to be obtained with a particular variety and the price per kg to be negotiated for that particular variety (Rajeswari and Suthendran, 2019; Jankovic et al., 2020). Then, we use these two variables in two ways. On the one hand, we employ them in the econometric models as farmers' individual characteristics in order to investigate if they determine variety selection and diversification. Introducing these variables as farmers' characteristics help understand if the yields obtained and prices negotiated by each farmer help determine the selection of the varieties grown. On the other hand, we employ these variables as variety-specific attributes. In order to introduce them as attributes, averages of these variables have to be taken since this empirical alternative aims at investigating whether expected yield and prices help determine variety selection in this region of Colombia. These averages are presented in Table 2 as attributes of each variety, and they are computed as the average values of yield and prices obtained by the farmers that cultivated these varieties in Santander. These averages can be considered as variety-specific attributes because they do not incorporate the influence of growing practices or market power exercised by particular farmers in the market.

## 4 Methodology

### 4.1 Variety selection

In a revealed-preference model, farmers' preferences for common beans are defined in terms of the attributes of the varieties available for selection (Lancaster, 1966). As preferences are not observed empirically, they are derived from farmers' choices of common bean varieties. This methodology is similar to the one employed in stated-preference models, where farmers' preferences are derived from their hypothetical choices of varieties. The difference between the two models is that in stated-preference experiments, farmers can compare all varieties under analysis and select those that attract them the most, after having considered their advertised attributes. In contrast, a revealed-preference model derives a ranking of preference for attributes from the varieties selected to be grown during the production cycle under analysis. In the latter case, farmers do not compare attributes and provides an indirect ranking by selecting a hypothetical set of varieties, but a preference ranking is derived from the selected varieties and the non-selected ones (Louviere et al., 2000). This procedure is less comprehensive, even though it allows a determination of the effect of some attributes on variety selection.

As a result, the choice of varieties by farmer *i* is modelled as maximizing a random utility function, *V*_*i*, *j*_, (McFadden, 1974) that is defined in terms of the set of attributes, *Z*_*j*_, provided by variety *j*, farmer i's socioeconomic characteristics, *X*_*i*_, and an error term *ε*_*i*, *j*_, which reflects unobserved idiosyncrasies of taste. Then, the indirect random utility of farmer *i* can be modelled as:

(1) $$V_{i,j}=\alpha^{\prime }Z_{j}+\beta \prime X_{i}+\varepsilon_{i,j}\;$$

where *α* is a vector of coefficients to be estimated, including an alternative specific constant, which is associated with the attributes of the varieties considered, and *β* is a vector of coefficients to be estimated, which is associated with farmer *i*'s socioeconomic characteristics. When the price of the variety *j* is used as one of its attributes, the estimated values of *α* can be used to determine farmer *i*'s economic valuation for each attribute. Louviere et al. (2000, pages 34–55) explain in detail the theoretical model and the estimation method for Eq. (1).

A key assumption to estimate Eq. (1) by an alternative-specific conditional logistic regression model is that the probability of a particular variety being chosen is independent of irrelevant alternatives (IIA). This assumption usually breaks down when some of the attributes included in *Z*_*j*_ are random, and decision-making is heavily influenced by this variation in attributes. This situation usually occurs when the attribute of a variety is randomly assigned, such as whether a variety is bio-fortified or drought-resistant by a genetic modification that does not change the other attributes of the same variety. In this case, the selection of a particular variety may be dependent upon the random assignment of that attribute, which implies that the selection of this variety is not independent of irrelevant alternatives. As in a revealed-preference model this interaction among attributes is rather rare since farmers grow the varieties they consider the best for them and their decision is not influenced by the presentation of systematic information on varieties and attributes, Eq. (1) can be estimated using an alternative-specific conditional logistic regression model, which assumes that *ε*_*i*, *j*_ follows a multinomial logistic distribution that is independently distributed across individuals and varieties.

We can create a vector^5^ *Y*_4528*x*1_ that captures the selection of varieties made by all 566 farmers in the sample. We can insert in the first eight rows the selection made by farmer 1, in the following eight rows the selection made by farmer 2, and so on. The first row for each farmer refers to whether that farmer cultivated Calima, the second row refers to Froilan, the third row to Corpoica Jiji, the fourth to Zaragoza, the fifth to Cargabello, the sixth to Radical, the seventh to Bola Roja, and finally the eighth row refers to Cargamanto Rojo. Hence, *Y*_4528*x*1_ defines a categorical variable with 8 categories. Using this vector, we can run a multinomial logistic regression model (MNL), which estimate the following probabilities using a maximum likelihood estimation method:

(2) $$P\left(Y=v_{j}\right)=\frac{e^{Z_{j}\alpha +\mathrm{X\beta}}}{\sum_{j=1}^{8}Z_{j}\alpha +\mathrm{X\beta}}$$

where 8 refers to the total number of categories in the vector *Y*, *Z*_*j*_ is a 4528xVA matrix associated with the number of attributes considered in the analysis, and X is a 4528xIC matrix associated with the number of socioeconomic characteristics considered in the model. VA and IC are related to the attributes of Table 2 and the socioeconomic characteristics of Table 3, respectively. *α* and *β* are two vectors to be estimated that are associated with the attributes and socioeconomic characteristics considered in the analysis, and *v*_*j*_ refers to variety *j*, ∀*j* = {1, 2, 3, 4, 5, 6, 7, 8}, which is associated with the ID of each variety as presented in Table 2. The estimation method is explained in detail by Louviere et al. (2000, 66) and Greene (2003, 720–763). For a MNL regression model, it is common to report the relative-risk ratios (RRR) instead of the estimated coefficients since the units of these estimations do not have a straightforward interpretation. These ratios are encountered by taking one category of *Y* as the base and expressing all estimated coefficients relative to that base. As a result, the RRR of an explanatory variable *x*_*s*_ measures whether a marginal change in *x*_*s*_ increases more the probability of choosing the variety used as a comparison group relative to the probability of choosing the variety used as the comparison group, or vice versa. An RRR larger than 1 indicates that a marginal increase in *x*_*s*_ increases more the probability that farmers choose the comparison variety than the base variety. In turn, an RRR smaller than 1 indicates that a marginal increase in *x*_*s*_ increases more the probability that farmers choose the base variety than the comparison variety. Finally, an RRR equal to 1 means that a marginal increase in *x*_*s*_ increases in the same proportion the probability that farmers choose both the comparison and base varieties.

### 4.2 Variety diversification

Variety diversification refers to the number of common bean varieties grown by farmers in the same production cycle. This decision is usually modelled using a random utility framework (Asfaw et al., 2013a, Asfaw et al., 2013b; Khonje et al., 2015). A farmer will grow two varieties of common beans instead of one when the difference between the utility of growing two varieties is larger than the utility of growing only one variety. Similarly, a farmer will grow three varieties when the utility of growing three varieties is larger than both the utility of growing two varieties and the utility of growing only one. Thus, the random utility of growing *s* number of varieties, *V*_*i*, *s*_, can be expressed as:

(3) $$V_{i,s}=\beta \prime X_{i}+\varepsilon_{i,s}\;$$

where *X*_*i*_ is farmer *i*'s socioeconomic characteristics hypothesised to help explain the utility derived from growing s number of varieties, *β* is a vector of coefficients to be estimated, *ε*_*i*, *s*_ is an error term capturing unobserved factors that also help explain *V*_*i*, *s*_, and *s* ∈ {1, 2, 3} is the number of varieties grown by farmer *i*. By definition, *V*_*i*, *s*_ is a latent variable. Defining *Y*_*i*_ as a categorical variable that captures the number of varieties grown by farmer *i*, the relationship between *Y*_*i*_ and *V*_*i*, *s*_ can be expressed as:

(4) $$\left\{\begin{array}{ccc} Y_{i}=1 & \mathrm{if} & V_{i,1}>V_{i,2}\;\mathrm{and}\;V_{i,1}>V_{i,3}\;\\ Y_{i}=2 & \mathrm{if} & V_{i,2}>V_{i,1}\;\mathrm{and}\;V_{i,2}>V_{i,3}\\ Y_{i}=3 & \mathrm{if} & V_{i,3}>V_{i,1}\;\mathrm{and}\;V_{i,3}>V_{i,2} \end{array}\right)$$

If this relationship can be modelled as an ordered categorical variable, it is expected that the following strong relationship holds:

(5) $$\left\{\begin{array}{ccc} Y_{i}=1 & \mathrm{if} & V_{i}<0\;\\ Y_{i}=2 & \mathrm{if} & 0<V_{i}>\mu^{1}\\ Y_{i}=3 & \mathrm{if} & \mu^{1}<V_{i}<\mu^{2} \end{array}\right)$$

where *V*_*i*_ = *β* ′ *X*_*i*_ + *ε*_*i*_ is the indirect utility obtained by farmer *i* from cultivating the selected variety, *μ*^1^ and *μ*^2^ are two values to be estimated jointly with *β*, which are defined in the domain of utility. The model in eq. (5) can be estimated using an ordered logistic regression model. In this case, it is also customary to report the RRR, which are always interpreted relative to the base category, *Y*_*i*_ = 1.

## 5 Results

### 5.1 Variety selection

Table 4 presents the econometric estimations for the alternative-specific conditional logistic regression model. All these regressions have statistically significant Wald tests, implying that all regressors used are statistically significant to explain the variability of variety selection. We run several econometric models with different combinations of attributes and socioeconomic factors, and the results presented have the smallest Bayesian Information Criterion (BIC) among all the regressions run. Three results are worth noticing on Table 4. First, the number of days to flowering and the price per kilogram of beans are the two most important attributes of common beans for this farming community. In particular, varieties with smaller vegetative phases and larger prices per kilogram are more demanded by farmers than varieties with larger vegetative phases and smaller prices per kilogram. This implies that the attributes of colour, size, and shape are not important determinants of bean selection in this region of Colombia. The reason for this result lies in the fact that almost all varieties grown in the region have a red colour, have a middle to large size and a similar shape. This reduced variability in the attributes considered may be due to the attributes demanded by consumers. If this is the case, all physical attributes are taken as given by Santander common bean producers, and the only attributes that affect their selection of varieties are those associated with the length of the duration of the crop and the economic reward obtained per kilogram sold. It is worth noting that days to flowering is only statistically significant at 10%. This result may be explained by the low variability in the number of days to flowering considered. In the sample of varieties analysed, 5 varieties have less than 100 days to flowering and only 3 have 100 days or more to flowering. This low variability may affect the estimated standard errors associated with the number of days to flowering, which in turn affect the efficiency with which the model determinates the significance of the corresponding estimated parameter. Even though this low variability, the model is capable of weakly identifying days to flowering as a driver behind variety selection in this region of Colombia.

**Table 4:** Relative Risk Ratios (RRR) for the Alternative-Specific Conditional Logistic Regression Model for the Common Bean Phenotypes grown in Santander, Colombia (8 Alternatives and 566 Cases).

Variables	Comparison Group/Base Group													
Bean Attributes	2/1^a^	3/1	4/1	5/1	6/1	7/1	8/1	3/2	4/2	5/2	6/2	7/2	8/2	4/3
days to flowering	0.68*	0.68*	0.68*	0.68*	0.68*	0.68*	0.68*	0.68*	0.68*	0.68*	0.68*	0.68*	0.68*	0.68*
price/kg (avg.)	1.05***	1.05***	1.05***	1.05***	1.05***	1.05***	1.05***	1.05***	1.05***	1.05***	1.05***	1.05***	1.05***	1.05***

Farm Specific Variables														
female importance (females - males)	0.77	0.99	0.92	1.10	1.07	3.36*	0.44***	1.28	1.20	1.43**	1.38**	4.37**	0.57***	0.93
household head age	0.95**	1.03	0.94***	0.97	0.96**	1.04	1.01	1.09***	1.00	1.03	1.02	1.09*	1.07	0.91***
household head's years of education	1.00	1.34*	0.86	0.93	0.95	1.51	0.79**	1.34*	0.86	0.93	0.95	1.51	0.79**	0.64***
number of farmers living in the farm	0.78	4.93	0.98	0.99	1.09	72.51*	2.43***	6.32	1.25	1.28	1.40	93.18*	3.13***	0.20
farm area (ha)	1.06	1.38	1.19	1.19	1.34	0.35	0.56*	1.3	1.12	1.12	1.26*	0.33	0.52***	0.86
number of crops cultivated	5.83***	2.33	4.11***	3.90***	3.17**	17.82*	5.53***	0.40**	0.71	0.67*	0.54***	3.06	0.95	1.76
elevation (masl)	1.00	0.99***	0.99***	1.00	1.01	1.03**	1.01	0.99***	0.99***	1.00	1.01**	1.03*	1.02	1.00
distance (km)	0.99	2.45***	1.19	1.02	1.12	0.03	0.92	2.47***	1.20	1.03	1.13	0.03	0.93	0.49***
number of inhabitants in the farm	0.97	0.16**	1.03	0.98	0.79	0.37	0.52***	0.17**	1.06	1.01	0.82	0.39	0.54***	6.29**
yield/ha*certified	0.98***	0.99***	1.00	0.98***	1.00	0.99***	1.01*	1.01***	1.02***	1.00	1.02***	1.01**	1.02***	1.01***
distance*aqueduct	1.54***	0.02***	0.95	1.13	1.38***	0.89	1.54***	0.09***	0.62***	0.73***	0.89	0.58	1.00	63.19***


Variables	Comparison Group/Base Group													
Bean Attributes	5/3	6/3	7/3	8/3	5/4	6/4	7/4	8/4	6/5	7/5	8/5	7/6	8/6	8/7
days to flowering	0.68*	0.68*	0.68*	0.68*	0.68*	0.68*	0.68*	0.68*	0.68*	0.68*	0.68*	0.68*	0.68*	0.68*
price/kg (avg.)	1.05***	1.05***	1.05***	1.05***	1.05***	1.05***	1.05***	1.05***	1.05***	1.05***	1.05***	1.05***	1.05***	1.05***

Farm Specific Variables														
female importance (females - males)	1.12	1.08	3.41	0.45	1.20	1.16	3.66**	0.48***	0.97	3.05*	0.40***	3.16*	0.41***	0.13***
household head age	0.94**	0.93**	1.01	0.98	1.03*	1.02	1.10*	1.08	0.99	1.07	1.05	1.08*	1.06	0.98
household head's years of education	0.70**	0.71**	1.13	0.59***	1.09	1.11	1.76*	0.92	1.02	1.62	0.85*	1.59	0.83**	0.52**
number of farmers living in the farm	0.21	0.22	14.72	0.49	1.02	1.12	74.34*	2.49***	1.10	73.01*	2.45***	66.68*	2.24***	0.04
farm area (ha)	0.86	0.97	0.25	0.40***	1.00	1.13	0.29	0.47***	1.13	0.29	0.47***	0.26	0.42***	1.61
number of crops cultivated	1.67	1.36	7.65	2.37	0.95	0.77	4.34	1.35	0.82	4.57	1.42	5.63	1.75	0.31
elevation (masl)	1.01***	1.01***	1.04**	1.02**	1.01***	1.01***	1.04**	1.02**	1.01***	1.03**	1.02	1.03*	1.01	0.98**
distance (km)	0.42***	0.46***	0.02	0.38***	0.86	0.94	0.03	0.77	1.11	0.03	0.91	0.03	0.82	28.04
number of inhabitants in the farm	5.97**	4.83**	2.29	3.17	0.95	0.77**	0.36	0.51***	0.81**	0.38	0.53***	0.47	0.66***	1.38
yield/ha*certified	0.99***	1.01***	0.99*	1.01***	0.98***	1.00	0.99***	1.01***	1.02***	1.01	1.02***	0.99***	1.01***	1.01*
distance*aqueduct	75.27***	91.48***	59.09***	102.35***	1.19**	1.45***	0.94	1.62***	1.22***	0.79	1.36**	0.65	1.12	1.73

***Statistically significant at 1%; ** statistically significant at 5%; * statistically significant at 10%.

^a^To obtain the RRR for the inverse relationship, divide 1 by the estimated coefficient in the table (i.e., the coefficient of female importance, for instance, for 1/2 is equal to 1/0.77 = 1.30).

Second, we created two variables by interacting the variables of certification with yield per hectare and the variables of distance with access to aqueduct. We introduced these variables for two reasons. On the one hand, using the variables of certification and access to aqueduct without interacting them generate a multicollinearity problem with the other regressors because these variables do not vary too much across individuals. On the other hand, interacting these variables with other variables, such as yield and distance, solve the problem of multicollinearity, which allows having some insight about the effect of certification and access to aqueduct on variety selection. The interaction between certification and yield per hectare captures the effect of yield provided by certified seeds on variety selection and the interaction between distance and access to aqueduct captures the effect of access to a stable water source at a far distance from the municipality's urban centre on seed selection. The results shown on Table 4 indicate that the interacted variables are statistically significant explanatory variables of variety selection in Santander. Moreover, the interaction between yield per ha and certification helps determine 46 out of the 56 pairwise relationships that arise among the existing common bean varieties in the Santander market^6^ and the interaction between distance and access to aqueduct help determine 34. Finally, elevation and female importance are the two most important socioeconomic determinants of variety selection in this farming community. Elevation helps explain 38 out of 56 pairwise relationships of Table 4 and female importance helps explain 26.

The statistically significant variables of Table 4 generate a ranking of preference for common bean varieties. Table 5 summarises this ranking for all the variables that resulted statistically significant in Table 4. Table 5 is organized from the variable that explains the most to the one variable that explains the least the variability of variety selection. The results on Table 5 indicate that farmers tend to prefer Cargamanto Rojo (8) over the other varieties in the market when their seeds are certified. These results also indicate that farmers located at higher elevations tend to prefer Bola Roja (7) over the other varieties in the market. However, when Bola Roja (7) is compared with Cargamanto Rojo (8), farmers located at higher elevations prefer Cargamanto Rojo (8) than Bola Roja (8), even though elevation does not help determine the relationship between Cargamanto Rojo (8) and Calima (1), Froilan (2), Cargabello (5), and Radical (6). In turn, Bola Roja (7) also tends to be preferred by households mainly composed by women. However, female importance does not help determine the relationship between Bola Roja (7) and Corpoica JiJi (3). In turn, the interaction between distance and access to aqueduct does not help determine the relationship between Froilan (2), Radical (6) and Cargamanto Rojo (8). What this interaction shows is that farmers located farther away from the urban centres but with access to a stable water source prefers any variety over Corpoica Jiji (3). In contrast, Corpoica Jiji (3) tends to be preferred by older farmers and by farmers with more years of education. In addition, Froilan (2) tends to be preferred by farmers that cultivate several other crops in their farms. Hence, Froilan is the variety most preferred for intercropping. A final result worth noticing is the distance does not have a large explanatory power of variety selection in this region of Colombia. Distance only influences the relationship between Corpoica JiJi (3) and all other varieties, except Bola Roja: Farmers located farther away from the urban centres tend to prefer Corpoica JiJi (3) over the rest.

**Table 5:** Common Bean Variety Rankings Generated by (Statistically Significant) Explanatory Variables (organized from the most preferred to the least preferred common bean variety).

yield/ha*certified	a	8	1,4,6	3	7	2
	b	8	1,4,6	3	5,7	
elevation (masl)	a	7	1,6	3,4		
	b	7	6	2,5	3,4	
	c	8	7	3,4		
distance*aqueduct	a	2,6,8	1,4	3		
	b	2,6,8	1,5	3		
	c	2,6,7,8	3			
female importance (females - males)	a	7	1	8		
	b	7	5,6	2	8	
	c	7	4,5,6	8		
	d	6,8	5	4	3	
household head's years of education	a	3	1,2	8		
	b	3	4,5,6			
	c	7	4,8			
	d	5,6,7	8			
number of inhabitants in the farm	a	1,2,4,5	3,8			
	b	4,5	6	3,8		
household head age	a	1,3,7	2,4			
	b	1,3	6			
	c	3	5	4		
number of farmers living in the farm	a	7,8	1,2,4,5,6			
number of crops cultivated	a	2,4,7,8	1			
	b	2	3,5,6			
	c	2	5,6	1		
farm area (ha)	a	1,2,3,4,5	8			
	b	6	2	8		
distance (km)	a	3	1,2,4,5,6,8			

1 = Calima; 2 = Froilán; 3 = Corpoica JiJi; 4 = Zaragoza; 5 = Cargabello; 6 = Radical; 7 = Bola Roja; 8 = Cargamanto Rojo

### 5.2 Variety diversification

Table 6 presents the regression results for the ordered logistic regression model. This regression has a Wald test that is statistically significant, which implies that the regressors used help explain the variability of variety diversification at 1%. The results on Table 6 indicate that farmers tend to grow more than one variety of common beans during the same production cycle when there are more farmers living in the house. This implies that more family help leads to more variety diversification. In turn, variety certification also leads to variety diversification. Hence, farmers are willing to grow more than one variety of beans when at least one of the varieties grown has certified seeds. In turn, having a tractor helps producing common beans in the areas in which these machines can be used on the Andean mountains. Thus, having a tractor leads farmers to grow more than one variety in the same production cycle. In contrast, farmers located at farther distance from the urban centres tend to grow fewer varieties. Finally, the other variables included in the regression have a low or null explanatory power of variety diversification. This implies that neither price nor yield nor access to a stable water source helps determine variety diversification in this region of Colombia.

**Table 6:** Regression Results for the Ordered Logistic Regression Model (N = 566).

Variables	Estimations
yield/ha (kg)	1.01
price/kg (CO$)	1.02
female importance (females - males)	0.94
household head age	0.99
household head's years of education	1.05
number of farmers living in the farm	2.05***
farm area (ha)	1.18
number of crops cultivated	0.8
elevation (masl)	1
distance (km)	0.69**
number of inhabitants in the farm	0.76*
aqueduct (1 = access, 0 = not access)	2.12
certified (1 = certified, 0 = not certified)	5.44***
tractor (1 = owns a tractor, 0 = otherwise)	10.89***
R^2^	0.1387
Walt Test	47.71***

*Statistically significant at 1%, **Statistically Significant at 5%, *Statistically Significant at 10%

It is worth noting that neither yield per hectare or price per kg is an important driver behind variety diversification in this Colombian region. These results may be explained by the fact that farmers grow more than one variety not necessarily to obtain the largest yield per hectare or the largest price per kilogram, but as a strategy to minimize potential risks affecting seasonal yield and total income accruing from common bean production. In fact, the drivers behind common beans diversification identified in Table 6 indicate Santander farmers who grow more than one variety do it because either they have the installed capacity to grow more than one variety or are incentivised by a wholesaler to grow certified seeds, which usually have a smaller market size and are more costly to acquire. Hence, common bean diversification in this region of Colombia seems to be driven by technical factors associated with the capacity of farmers to grow more than variety than by economic factors, such as price per kilogram or yield per hectare.

## 6 Discussion

Voluntary uptake of new phenotypes of common bean is expected to be determined by similar factors that determine farmers' demand for common beans currently in the market (Lambrecht et al., 2013a, Lambrecht et al., 2013b; Lambrecht et al., 2015; Assete et al., 2018). Previous research has found that the most popular attributes of common beans demanded by farmers are drought and disease tolerance, yield, taste, and cooking time (Graf et al., 1991; Sperling et al., 1993; Odendo et al., 2004; Katungi et al., 2011a; Katungi et al., 2015). All these studies have been carried out in African countries where common bean production is important. No study has been done to identify the attributes that determine variety selection in Latin America. In this study, we performed the first identification of the determinants of variety selection in Colombia. This study identifies the length in vegetative state and the price per kg received as two of the most important variety-specific attributes that determine variety selection of common beans in the study area. Thus, varieties with a shorter vegetative phase and with a larger price per kg are more demanded by farmers in Santander. This study also shows that other attributes, such as colour, shape, and size, are not important determinants of variety selection in the study region. This result may be explained by the attributes demanded by final consumers in this region of Colombia since most of the varieties available in the market tend to have the same colour, shape, and size, which reduce its variability and explanatory power as determinants of variety selection in this region of Colombia.

Studies carried out in African countries also identify farmer-specific factors that determine variety selection (Katungi et al., 2011a; Katungi et al., 2015). None of the studies performed in Africa has emphasised the role of farms' elevation or seed certification on variety selection. In this study, we identify four farmer-specific determinants of variety selection in the study region: Elevation, seed certification, females' influence on production decisions and access to a stable water source when the farm is located farther away from the urban centres. Elevation is an important determinant of agricultural production in Colombia (IDEAM, 2013; FENALCE, 2020). This variable is also an important determinant of common bean breeders' climate change perception (Botero et al., 2020). In this study, we find that farms' elevation helps explain variety selection as well. Our econometric results indicate that farmers located at higher elevations prefer to grow Bola Roja, while farmers located at lower elevations prefer to cultivate either Zaragoza or Corpoica JiJi. In turn, seed certification is found to be an important determinant of the demand for Cargamanto Rojo. Moreover, farmers who tend to demand a certified seed also tend not to grow either Froilan or Bola Roja. In contrast, farms where there are more females than men tend to grow Bola Roja and avoid growing Cargamanto Rojo. Finally, farmers who have access to the aqueduct and are located further away from the urban centres prefer to cultivate Froilan, Radical, or Cargamanto Rojo than the other varieties in the market.

Common bean variety diversification is also studied in African countries (Onzima et al., 2019). This literature emphasises the role of household composition and capital goods on variety diversification. Our econometric results indicate that common bean variety diversification in Santander is determined by the number of family workers living in the farm and tractor ownership. In other words, farmers who count with more help from their family and have a tractor tend to grow more than one variety of beans in the same production cycle. In contrast, farmers located farther away from the urban centres tend to grow only one variety of beans. The latter result may be explained by the fact that farmers located farther away from urban centres have larger transportation costs, which influences the number of varieties grown by them.

The results of this study show that agricultural companies and seed suppliers can utilise farms' elevation and household composition as two important factors to define engagement strategies to maximize the uptake of particular bean varieties. In particular, seed suppliers attempting to introduce climbing beans could focus on high-elevation farms since these farmers are willing to grow this type of beans. In addition, seed suppliers attempting to introduce certified varieties could focus on farmers located at farther distance from the urban centres since these farmers are willing to grow certified seeds. However, if the aim is to incentivise common bean variety diversification, seed suppliers can focus on farmers located closer to urban centres or on farmers who receive more help from family members to grow beans since these farmers tend to grow more than one variety in the same production cycle.

## 7 Conclusions

The ENSO phenomenon has an important impact on common bean production areas in Colombia. This phenomenon affects common bean production areas asymmetrically. Low-elevation farms are more affected by droughts and high temperatures, whereas high-elevation farms are mostly affected by extreme weather variations and unpredictable seasons. This elevation-driven weather variation has a great effect on common bean productivity in Colombia. In order to help Colombian farmers to adapt to climate change, Colombian agricultural companies have developed common beans' adaptation programs. These programs aim at developing phenotypes that are resilient to abiotic and biotic stresses generated by ENSO and climate change. Most of the varieties developed so far have had a low uptake by Colombian farmers. One of the reasons is that the development of common beans has been based on experts' opinion about Colombian farmers' necessary adaptations, disregarding farmers' opinions and farmer-specific drivers behind the demand of particular common bean phenotypes. In the literature, it has been argued that the uptake of the common bean varieties under development is expected to depend on similar factors that determine the demand for the common bean varieties currently in the market. This paper provides the first identification of the factors that determine variety selection and diversification in one of the most important common bean production areas in Colombia — the department of Santander.

Our econometric results indicate that elevation is one of the most important determinants of variety selection in Santander. Farmers located at higher elevations tend to demand the varieties known as Bola Roja and Cargamanto Rojo; two varieties that are recognised to perform relatively well at high elevations. In contrast, farmers located at low elevations tend to prefer traditional varieties, such as Zaragoza. These traditional varieties are usually developed or maintained by farmers themselves, and they continue to be grown because farmers prefer to rely on varieties that they know. Another important determinant of variety selection is seed certification. Farmers tend to increase the demand of Cargamanto Rojo when the seeds utilised are certified. This result implies that farmers are willing to cultivate a climbing variety when the seeds utilised are certified by a farmers' association or specialised seed producer. Finally, household composition is another important driver behind variety selection. Households where female have an important influence on production decisions tend to prefer cultivating Bola Roja, whereas households mainly controlled by males tend to prefer cultivating varieties that are harder to cultivate but that provide a larger yield, such as Cargamanto Rojo.

We also investigate the determinants of common bean variety diversification in Santander. Our econometrics result show that farmers who have several family members working with them in the farm and those owning a tractor tend to grow more than one variety in the same production cycle. This implies that farmers with more physical capital and with a larger household composition tend to have a larger common bean variety diversification. In contrast, farmers who are located farther away from the main urban centres tend to only grow one variety of common beans per production cycle. This implies that farmers with larger transportation costs due to the location of their farms relative to the main urban centres tend to have a reduced common bean variety diversification.

Seed suppliers and extension service providers can use these drivers to increase common bean diversification or maximize the uptake of particular common bean phenotypes. In addition, companies that have common bean breeding programs can utilise these determinants to develop new common bean phenotypes. In that sense, if these companies want to increase the uptake of climbing common beans that are also climate-adapted should focus their commercial programs on farmers located at higher altitudes since these farmers tend to have a higher uptake of climbing varieties. In turn, if these companies want to maximize the uptake of climate-adapted beans, they should develop varieties similar to Zaragoza or Corpoica JiJi since these varieties tend to be grown by farmers located at low elevations, which are at the same time the most affected by droughts and high temperatures. Following this strategy, common bean developers can design and deliver more cost-effective engagement and commercial programs with communities of interest.

## Declaration of Competing Interest

The authors declare that they have no known competing financial interests or personal relationships that could have appeared to influence the work reported in this paper.

## Footnotes

1 A thermal floor is defined as the climate of a particular elevation along the mountain range of the Colombian Andes.

2 In CIAT categorization, Category I defines a bush plant with a determine growing habit and Category III defines a climbing plant with an indeterminate growing habit (Schoonhoven and Voysest, 1991; page 146).

3 This categorization may be found in Schoonhoven and Voysest (1991), pages 126–144.

4 We also introduced certification as one of the attributes of common beans. Based on the varieties developed by AGROSAVIA and FENALCE, we defined certification as a variety-specific attribute by putting a 1 to those varieties known to have been developed by these institutions (Calima, Froilan, Corpoica JiJi, Bola Roja and Cargamanto Rojo). We introduced two sets of dummies for this attribute, one dummy considering Calima as a certified variety and the other dummy not considering Calima as a certified variety. None of these variety-specific dummies are statistically significant.

5 Eight varieties of common beans are grown in the study area and there are 566 households in the sample, which implies that 4528 = 8*566.

6 Each variety can be used both as a base category and as a comparison category. Hence, each variety is used as a base for 7 relationships and there are 8 varieties (56 = 7*8).
